# Effects of a vitamin and mineral bolus on beef heifer feedlot performance, feeding behavior, carcass characteristics, and liver mineral concentrations^1^

**DOI:** 10.1093/tas/txaa027

**Published:** 2020-04-13

**Authors:** Kacie L McCarthy, Sarah R Underdahl, Carl R Dahlen

**Affiliations:** Department of Animal Sciences, North Dakota State University, Fargo, ND

**Keywords:** feeding behavior, feedlot heifers, liver mineral, mineral and vitamin bolus

## Abstract

Crossbred beef heifers (*n* = 23; initial body weight [BW] = 370 ± 12 kg) housed at the North Dakota State University Beef Cattle Research Complex in Fargo, ND, were used to evaluate the effects of a 250-d slow-release vitamin and mineral bolus on feedlot performance, feeding behavior, carcass characteristics, and liver mineral concentrations. Heifers were assigned to one of two treatments: 1) received no supplemental mineral or vitamin (CON, *n* = 12) or 2) received two boluses on day 0 (minimum of 3,740 mg Ca, 16,456 mg Mg, 112 mg Na, 11,220 mg Cu, 2,995 mg I, 2,805 mg Mn, 505 mg Se, 48,620 mg Zn, 468 mg Co, 824,296 IU vitamin A, 173,102 IU vitamin D3, and 4,121 IU vitamin E bases on company analysis; Reloader 250 Mineral Bolus, Cargill Inc., Minneapolis, MN; MIN, *n* = 11). Heifers were fed a total mixed ration containing corn silage, grass hay, dried distillers grains with solubles, and dry-rolled corn (16.05% crude protein, 1.44 Mcal/kg NEg) with no added vitamin or mineral supplement. Feed intake and number and time of visits were recorded for each heifer using the Insentec feeding system (Hokofarm Group B.V., the Netherlands) during the feeding period. Liver biopsies were collected from heifers on days 0, 69, and 134 of the feeding period for analysis of mineral concentrations and analyzed as repeated measures. Heifers were slaughtered after 150 or 169 d on feed, and carcass characteristics were determined. Final BW, average daily gain, dry matter index, gain:feed, and carcass characteristics were not influenced (*P* > 0.19) by treatment. Control heifers visited feeders more but spent less time per visit and ate less per visit compared with MIN heifers (*P* < 0.03). No differences (*P* > 0.06) in liver mineral concentrations were observed between treatments, and concentrations of Se, Cu, Mo, Mn, and Co decreased (*P* < 0.05) over the feeding period. In this experiment, the slow-release vitamin and mineral bolus evaluated failed to increase liver mineral concentrations during the finishing period or influence heifer performance and carcass characteristics.

## INTRODUCTION

Diet alone often does not supply sufficient amounts of trace minerals; therefore, supplementation is necessary to optimize animal health and performance (NASEM, 2016). Research clearly has documented that intakes of free-choice minerals are variable among animals, with some cattle overconsuming or underconsuming supplements (Greene, 2000). Furthermore, inadequate trace mineral consumption can compromise reproduction, animal health, and animal growth (NRC, 2005; NASEM, 2016).

In confinement scenarios, liquid, loose dry, and pelleted dry supplements are easily fed as an ingredient in finishing diets, often with the additional vitamins provided to key classes of cattle (Samuelson et al., 2016). In range or pasture settings, however, delivery of supplemental mineral and vitamin formulations can be problematic because of the inconvenience of transporting large quantities of feed to remote grazing sites.

A long-acting (6-mo) reticulorumen trace mineral bolus developed in the United Kingdom (Cosecure; Telsol Ltd., Leeds, UK) containing Cu, Se, and Co has shown promise in increasing liver Cu and blood Se levels in range cows (Sprinkle et al., 2006). A recently developed bolus product was released in the United States (Reloader 250 Mineral Bolus, Cargill Inc., Minneapolis, MN) that targets a slow release over 250 d. A long-acting delivery of trace minerals could be advantageous for beef production in extensively managed systems and would eliminate variation in intake by ensuring a consistent supply over the payout period. Therefore, this study was designed as a model to evaluate the slow-release bolus in a confinement scenario prior to testing the bolus in extensive environments. The objectives of this study were to evaluate the effects of a mineral and vitamin bolus on heifer feedlot performance, feeding behavior, carcass characteristics, and liver mineral concentrations. We hypothesized that a mineral and vitamin bolus would increase liver mineral concentrations and positively affect performance and carcass characteristics.

## MATERIALS AND METHODS

All animal procedures were approved by the Institutional Animal Care and Use Committee at North Dakota State University (NDSU; A18021).

### Animals, Diet, Body Weights, and Intake Measurements

Twenty-three crossbred beef heifers (initial body weight [BW] = 370 ± 12 kg) were housed at the NDSU Beef Cattle Research Complex in Fargo, ND. Heifers were weighed before feed delivery for two consecutive days at the beginning of the experiment and every 28 d throughout the feeding period. Heifers were assigned to one of two treatments: 1) received no supplemental mineral or vitamin bolus (CON, *n* = 12), or 2) received two boluses on day 0 (minimum of 3,740 mg Ca, 16,456 mg Mg, 112 mg Na, 11,220 mg Cu, 2,995 mg I, 2,805 mg Mn, 505 mg Se, 48,620 mg Zn, 468 mg Co, 824,296 IU vitamin A, 173,102 IU vitamin D3, and 4,121 IU vitamin E bases on company analysis; Reloader 250 Mineral Bolus, Cargill Inc., Minneapolis, MN (Table 1); MIN, *n* = 11). Heifers were fed a total mixed ration (TMR) that consisted of corn silage, grass hay, dried distillers grain plus solubles, and dry-rolled corn (Table 2; 1.44 Mcal/kg NEg) with no added vitamin or mineral supplement. Diets were offered for ad libitum intake, and heifers had free access to water. Ingredients were sourced as single lots and used over the duration of the experiment.

**Table 1.** Chemical composition slow-release bolus based on company-guaranteed analysis, total amount of mineral and vitamin administered, and daily availability over payout period^*a*^

	Bolus composition		Total administered^*b*^
Item	Min	Max	Min
Minerals^*c*^	mg/kg		mg
Ca	20,000	25,000	3,740
Mg	88,000	—	16,456
Na	600	2,000	112
Cu	60,000	—	11,220
I	15,800	—	2,955
Mn	15,000	—	2,805
Se	2,700	—	505
Zn	260,000	—	48,620
Co	2,500	—	468
	IU/kg		IU
Vitamins^*d*^			
Vitamin A	4,410,000	—	824,296
Vitamin D3	926,100	—	173,102
Vitamin E	22,050	—	4,121

^*a*^Reloader 250 Mineral Bolus, Cargill Inc., Minneapolis, MN. Each heifer received two boluses with a recommended payout rate of 250 d. One bolus weighs approximately 93.5 g.

^*b*^Amount of mineral and vitamin delivered based on administration of two boluses per heifer each weighing 93.5 g.

^*c*^Ingredients: zinc oxide, copper sulfate, magnesium oxide, sodium selenite, manganous oxide, calcium iodate, zinc amino acid chelate, cobalt carbonate, and calcium carbonate.

^*d*^Ingredients: vitamin A acetate, vitamin E supplement (proprietary), and vitamin D3 supplement (proprietary).

**Table 2.** Dietary ingredient and nutrient composition of TMR diet provided to finishing heifers

Item	DM basis
Ingredient	%
Corn silage	10.0
Grass hay	5.0
Corn	60.0
Dried distillers grains with solubles	20.0
Premix^*a*^	5.0
Nutrient analysis	%
DM	72.59
Ash	4.69
CP	16.05
N	2.57
NDF	24.78
ADF	9.35
Ether extract	3.25
Ca	0.65
P	0.45
Mineral analysis	mg/kg
Fe	130
Cu	4.4
Zn	32.7
Mo	0.6
Mn	23.7

^*a*^Premix composition: 28.6% fine ground corn, 36% limestone, 4.8% salt, 0.4% Monensin premix (176.4 g/kg), 0.2% Tylosin premix (88.2 g/kg), and 30% urea.

Heifers received a radio frequency identification tag in the right ear before the experiment that allowed access to the Insentec feeding system (Hokofarm Group B.V., the Netherlands). Feed intake and behavior were summarized by day for each individual heifer (Montanholi et al., 2010; Swanson et al., 2014) as follows: events (number of bunk visits and meals daily), eating time (minutes; per visit, per meal, and per day), and feed intake (kilograms; per visit, per meal, and per minute). A visit was defined as each time the feeding system detected a heifer at a bunk. A meal was defined as eating periods that might include short breaks separated by intervals not longer than 7 min (Montanholi et al., 2010; Swanson et al., 2014).

### Feed Analysis

Diet TMR samples were collected weekly throughout the experiment and composited over the feeding period. Weekly samples were dried in a 55-°C oven and ground to pass a 2-mm screen. The sample was analyzed at the NDSU Nutrition Laboratory for dry matter (DM), crude protein (CP), ash, N (Kjehldahl method), Ca, P, and ether extract (EE) by standard procedures (AOAC 1990). Multiplying N by 6.25 determined CP calculation. Neutral detergent fiber (NDF) and acid detergent fiber (ADF) concentrations were determined by the modified method of Van Soest et al. (1991) using a fiber analyzer (Ankom Technology Corp., Fairport, NY). The TMR diet was also analyzed for Cu, Zn, Co, Mo, Fe, S, and Se using inductively coupled plasma optical emission spectroscopy by the Veterinary Diagnostic Laboratory at Michigan State University.

### Liver Mineral Collections

Liver biopsies were collected from heifers on days 0, 69, and 134 of the feeding period to measure concentrations of trace minerals. Heifers were restrained in a squeeze chute and the hair between the 10th and 12th ribs was clipped with size 40 blades (Oster; Sunbeam Products Inc., Boca Raton, FL). Liver biopsy samples (approximately 20 mg) were collected using the method of Engle and Spears (2000) with the modifications that all heifers were given a subcutaneous 3-mL injection of Lidocaine Injectable-2% (MWI, Boise, ID) at the target biopsy site. An imaginary line is drawn from the tuber coxae (hook) to the elbow. At the intersection with a line drawn horizontally from the greater trochanter, a stab incision was then made between the 10th intercostal space. A core sample of the liver was taken via the Tru-Cut biopsy trochar (14 g; Merit Medical, South Jordan, UT).

The liver sample was placed on ashless filter paper (Whatman 541 Hardened Ashless filter paper, GE Healthcare Bio-Sciences, Pittsburg, PA) and then stored in tubes designed for trace mineral analysis (potassium Ethylenediaminetetraacetic acid; Becton Dickinson Co., Franklin Lakes, NJ) and stored at −20 °C until further analysis. After obtaining liver biopsies, a staple (Disposable Skin Staple 35 Wide; Amerisource Bergen, Chesterbrook, PA) and topical antibiotic (Aluspray; Neogen Animal Safety, Lexington, KY) was applied to the surgical site and an injectable NSAID (Banamine; Merck Animal Health, Madison, NJ) was given intravenously at 1.1 mg/kg of BW. Liver samples were shipped frozen on ice packs to the Veterinary Diagnostic Laboratory at Michigan State University and were evaluated for concentrations of minerals using inductively coupled plasma mass spectrometry.

### Carcass Characteristics and Bolus Recovery

Heifers were fed until visually observed to be finished (approximately 12 mm subcutaneous [s.c.] fat thickness at the 12th rib) with harvest groups processed at 150 (*n* = 16; CON = 9 and MIN = 7) and 169 (*n* = 7; CON = 3 and MIN = 4) days on feed. Final 2-d BWs were recorded prior to heifers being transported approximately 534 km to Dakota City, NE, to a commercial abattoir. Hot carcass weight (HCW) data were obtained following animal slaughter, whereas marbling score, back fat, longissimus muscle area, and yield grade were determined after carcasses were chilled for 24 h. Dressing percentages were calculated using HCW and final weights.

Upon harvest, the rumen and its contents were collected from a subset of heifers (*n* = 4, 8 boluses) for bolus recovery. The rumen was opened and contents sorted and boluses were removed from the rumen. Upon collection, boluses were rinsed clean in cold water, air dried for 48 h, and then weighed.

### Statistical Analysis

Data were analyzed as a completely randomized design using the MIXED procedure of SAS (9.4, SAS Inst. Inc., Cary, NC) for effects of treatment on intake, behavior, and carcass characteristics. The Kenward–Roger approximation was used to determine the denominator degrees of freedom for the tests of fixed effects. Carcass characteristics were tested initially for the effects of treatment, harvest group, and all interactions but found harvest group and the interaction to be nonsignificant and, subsequently, removed from the final model. Liver mineral concentrations were also analyzed using the MIXED procedure and Kenward–Roger approximation to determine the denominator degrees of freedom for the tests of fixed effects. The model statement used contained the effects of treatment, time, and all interactions. The specified term for the repeated statement was time, and heifer was included as the subject. The covariance structure used was variance components by providing the smallest Akaike information criterion for all variables analyzed. For all analyses, individual heifer was the experimental unit and results are reported as least square means with significance set at *P* ≤ 0.05.

## RESULTS AND DISCUSSION

### Liver Mineral Concentrations

No treatment × time interactions (*P* ≥ 0.70) or main effects of treatment (*P* ≥ 0.21) were observed for concentrations of Se, Fe, Cu, Zn, Mo, or Mn in the liver (Table 3). In contrast, 248 kg steers receiving a single Reloader 250 bolus had elevated concentrations of Se beginning at day 120 after administration compared with untreated steers (Jackson et al., 2020). The authors postulated that treatment divergence 120 d after bolus administration could have been due to reduced pH associated with a transition to high concentrate diets that occurred in the mid-point of their study. However, in the current experiment, heifers were on a finishing ration over the duration of the monitoring period and no divergence in liver mineral concentrations was observed.

**Table 3.** Liver mineral concentrations from heifers receiving vitamin and mineral bolus

	Treatment^*a*^									
	Day 0		Day 69		Day 134			*P*-value		
Item, µg/g	CON	MIN	CON	MIN	CON	MIN	SEM	TRT	Time^*b*^	TRT × Time
Se	1.89	1.87	1.64	1.71	1.53	1.55	0.08	0.75	0.0007	0.86
Fe	125	122	137	146	163	174	9	0.43	<0.0001	0.68
Cu	131	136	93	107	77	81	13	0.46	0.0002	0.91
Zn	104	107	102	111	101	104	6	0.27	0.73	0.86
Mo	3.95	3.86	3.40	3.53	3.54	3.47	0.14	0.93	0.005	0.70
Mn	8.92	9.26	7.95	8.43	8.18	8.54	0.37	0.21	0.05	0.98
Co	0.13	0.13	0.10	0.12	0.12	0.13	0.01	0.06	0.02	0.06

^*a*^Treatment: CON = heifers did not receive vitamin and mineral bolus; MIN = heifers received vitamin and mineral bolus containing a minimum of 3,740 mg Ca, 16,456 mg Mg, 112 mg Na, 11,220 mg Cu, 2,995 mg I, 2,805 mg Mn, 505 mg Se, 48,620 mg Zn, 468 mg Co, 824,296 IU vitamin A, 173,102 IU vitamin D3, and 4,121 IU vitamin E based on company analysis; Reloader 250 Mineral Bolus, Cargill Inc., Minneapolis, MN.

^*b*^Time: days 0, 69, and 134 were analyzed as repeated measures.

Concentrations of Co tended (*P* = 0.06) to be impacted by a treatment × time interaction. As indicated by the interaction, Co liver concentrations were similar at the start of the study and day 69 concentrations decreased and then returned to initial concentrations by the end of the feeding period. It is important to note that concentrations of Se, Cu, Mo, and Mn decreased (*P* < 0.05) over time on feed, whereas concentrations of Fe increased (*P* < 0.001). Analysis of our diet during the evaluation period (Table 2) indicated that concentrations of Fe, Zn, and Mn met the requirements of 50, 30, and 20 mg/kg, respectively, but the diet was deficient in Cu (requirement of 10 mg/kg; NASEM, 2016). By the end of the finishing period, liver concentrations of Cu were considered marginal (marginal = 33–125 μg/g DM; Kincaid, 2000), and concentrations of Mn were marginal throughout the experiment (marginally deficient = 7–15 μg/g DM; Kincaid, 2000). However, similar liver Mn concentrations have been reported in finishing steers (Niedermayer et al., 2018) and growing heifers (Hansen et al., 2006). Administration of supplemental mineral via the slow-release bolus in the current experiment failed to increase liver concentrations of mineral over the course of the feeding period, resulting in marginal deficiencies of Cu and Mn in both CON and MIN heifers by the end of our monitoring period.

Over a payout period of 250 d, the slow-release bolus evaluated in the current report would theoretically provide 194.5 mg of Zn/d, 44.9 mg of Cu/d, 1.9 mg of Co/d, 11.2 mg of Mn/d, and 2.0 mg of Se/d. However, there were no differences among treatments for concentrations of respective minerals in samples of the liver. In contrast, a slow-release bolus delivering more daily mineral (156 mg of Cu/d, 5.9 mg of Co/d, and 3.4 mg of Se/d) over a 175-d period resulted in elevated concentrations of Cu in the liver and Se in whole blood (Sprinkle et al., 2006). In the current experiment, weight of boluses retrieved from a subset of heifers 169 d after administration in the current experiment was 78.4 ± 0.58 g of the original 93.5 g, indicating that, after 68% of payout period (169 of 250 d), a very large proportion of bolus weight was still retained (84%). Taken together, these data suggest that changes in liver mineral were not detectable because of lower daily dosage delivered compared with other reports (Sprinkle et al., 2006) and/or that, perhaps, the boluses evaluated failed to deliver mineral at levels equivalent to their theoretical daily deliveries as evidenced by the large proportion of weight retained in the recovered boluses.

### Feeding Behavior, Performance, and Carcass Characteristics

Control heifers visited the feeders more frequently (*P* = 0.01; Table 4) compared with heifers receiving the mineral bolus, which means that CON heifers were detected at the bunk more times than MIN heifers. However, the number of meals per day was not influenced (*P* = 0.96) by treatment. Heifers receiving the mineral bolus ate longer (*P* = 0.01) per visit than control heifers, but the amount of time heifers spent eating per meal and per day was not different (*P* > 0.49) between treatments. Heifers receiving the mineral bolus ate more per visit (*P* = 0.03) compared with control heifers, but we found no differences (*P* > 0.08) in eating rate per meal and eating rate per minute between treatments. It is not clear why mineral bolus heifers ate more per visit, nor is it clear why mineral bolus heifers ate longer per visit than control heifers. The divergence present among treatments on a per-visit basis was not observed on a per-meal assessment or in terms of overall dry matter index (DMI), time spent eating, or eating rates; the treatment groups ultimately ate the same amount of feed at the same overall rate and therefore, visit differences may be of little biological relevance.

**Table 4.** Feed intake and feeding behavior of heifers receiving vitamin and mineral bolus

	Treatment^*a*^			
Item	CON	MIN	SEM	*P*-value
Initial BW, kg	372	367	12	0.77
Final BW, kg	557	551	14	0.77
ADG, kg	1.23	1.23	0.05	0.93
DMI, kg	9.93	9.48	0.25	0.23
G:F, kg gain/kg feed	0.125	0.129	0.004	0.41
Events, per d				
Visits^*b*^	39.24	31.43	1.75	0.01
Meals^*c*^	8.24	8.22	0.29	0.96
Time eating, min				
Per visit	1.64	2.09	0.11	0.01
Per meal	7.91	8.14	0.37	0.66
Per day	61.24	63.93	2.62	0.49
Eating rate, kg				
Per visit	0.26	0.31	0.02	0.03
Per meal	1.25	1.21	0.04	0.51
Per min	0.157	0.142	0.01	0.08

^*a*^Treatment: CON = heifers did not receive vitamin and mineral bolus, or MIN = heifers received vitamin and mineral bolus containing a minimum of 3,740 mg Ca, 16,456 mg Mg, 112 mg Na, 11,220 mg Cu, 2,995 mg I, 2,805 mg Mn, 505 mg Se, 48,620 mg Zn, 468 mg Co, 824,296 IU vitamin A, 173,102 IU vitamin D3 and 4,121 IU vitamin E based on company analysis; Reloader 250 Mineral Bolus, Cargill Inc., Minneapolis, MN.

^*b*^A visit was defined as each time the Insentec system (Hokofarm Group B.V., the Netherlands) detected a heifer at a bunk.

^*c*^A meal was defined as eating periods that might include short breaks separated by intervals not longer than 7 min (Montanholi et al., 2010; Swanson et al., 2014).

Final BW did not differ between treatments (Table 4; *P* = 0.77). We found no differences (*P* = 0.93) in average daily gain (ADG) between treatments, with heifers gaining 1.23 ± 0.05 kg/d. No differences (*P* = 0.23) were observed in DMI between treatments, with heifers consuming 9.71 ± 0.25 kg/d. Feed efficiency (gain:feed [G:F]) also was not influenced (*P* = 0.41) by treatment and averaged 0.127 ± 0.004 kg/kg. In previous experiments, performance responses have been variable relative to trace mineral or Cu and Zn supplementation on finishing steer performance, with no effect on ADG, DMI, or G:F (Spears and Kegley, 2002; Ahola et al., 2005) versus nonsupplemented controls, whereas DMI decreased as Zn supplementation increased (Malcolm-Callis et al., 2000). Furthermore, Greene et al. (1988) found that steers fed either control or diets supplemented with zinc methionine or zinc oxide all had similar gains and intakes over a 112-d trial. Another contributing factor to our observations may have been the basal mineral concentrations in the diet. Feeding TM at NASEM recommended levels enhanced DMI in yearling steers compared with no supplemental TM, but ADG and G:F were not impacted unless steers were fed TM at levels greater than NASEM recommendations (i.e. levels recommended by feedlot consultants; Niedermayer et al., 2018). The lack of performance differences in the current experiment is not surprising given the lack of differences observed in liver mineral concentrations.

No differences (Table 5; *P* = 0.86) were observed between treatments for HCW, which averaged 341 ± 9 kg. No differences (*P* = 0.94) were observed between treatments for dressing percentage, which averaged 61 ± 0.004%. In addition, no differences (*P* > 0.38) were observed in back fat, longissimus area, yield grade, and marbling score between treatments (*P* ≥ 0.19). Carcass data resulting from the current experiment need to be interpreted with caution, however, because no detectable differences were observed in liver mineral concentrations.

**Table 5.** Carcass characteristics from heifers receiving vitamin and mineral bolus

	Treatment^*a*^			
Item	CON	MIN	SEM	*P*-value
HCW, kg	342	340	9	0.86
Dressing percentage	0.61	0.61	0.004	0.94
Marbling score^*b*^	584	523	33	0.19
Back fat, cm	1.48	1.31	0.13	0.38
Longissimus area, cm^2^	87.2	86.1	2.8	0.79
Calculated yield grade	2.85	2.83	0.20	0.95

^*a*^Treatment: CON = heifers did not receive vitamin and mineral bolus; MIN = heifers received vitamin and mineral bolus containing a minimum of 3,740 mg Ca, 16,456 mg Mg, 112 mg Na, 11,220 mg Cu, 2,995 mg I, 2,805 mg Mn, 505 mg Se, 48,620 mg Zn, 468 mg Co, 824,296 IU vitamin A, 173,102 IU vitamin D3, and 4,121 IU vitamin E based on company analysis; Reloader 250 Mineral Bolus, Cargill Inc., Minneapolis, MN.

^*b*^Marbling score: small = 400–499, modest = 500–599 and moderate = 600–699 (Emerson et al., 2013).

## CONCLUSIONS

Overall, our results indicate that finishing beef heifers using feeds sourced from the Northern Great Plains met dietary requirements for Fe, Zn, and Mn, with Cu falling below requirements in the diet provided. The inclusion of a slow-release vitamin and mineral bolus resulted in transient changes in heifer feeding behavior, which did not result in alterations in overall DMI, number of meals, or eating rate. Further, the slow-release mineral and vitamin boluses did not influence heifer performance or carcass characteristics and did not support or improve liver mineral concentrations over control heifers as concentrations of Se, Cu, and Mo decreased throughout the evaluation period.
